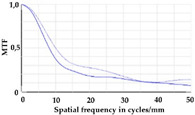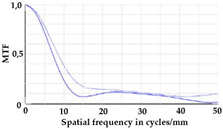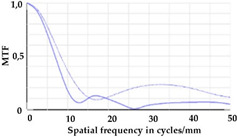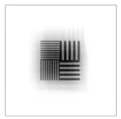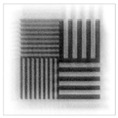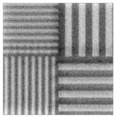# Enhancement of High-Resolution 3D Inkjet-Printing of Optical Freeform Surfaces Using Digital Twins

**DOI:** 10.3390/mi12010035

**Published:** 2020-12-30

**Authors:** Ingo Sieber, Richard Thelen, Ulrich Gengenbach

**Affiliations:** 1 Institute for Automation and Applied Informatics, Karlsruhe Institute of Technology, Hermann-von-Helmholtz-Platz 1, 76344 Eggenstein-Leopoldshafen, Germany; ulrich.gengenbach@kit.edu; 2 Institute of Microstructure Technology-KNMF, Karlsruhe Institute of Technology, Hermann-von-Helmholtz-Platz 1, 76344 Eggenstein-Leopoldshafen, Germany; richard.thelen@kit.edu

**Keywords:** digital twin, modeling, simulation, additive manufacturing, 3D inkjet-printing, freeform optics, varifocal optics

## Abstract

3D-inkjet-printing is just beginning to take off in the optical field. Advantages of this technique include its fast and cost-efficient fabrication without tooling costs. However, there are still obstacles preventing 3D inkjet-printing from a broad usage in optics, e.g., insufficient form fidelity. In this article, we present the formulation of a digital twin by the enhancement of an optical model by integrating geometrical measurement data. This approach strengthens the high-precision 3D printing process to fulfil optical precision requirements. A process flow between the design of freeform components, fabrication by inkjet printing, the geometrical measurement of the fabricated optical surface, and the feedback of the measurement data into the simulation model was developed, and its interfaces were defined. The evaluation of the measurements allowed for the adaptation of the printing process to compensate for process errors and tolerances. Furthermore, the performance of the manufactured component was simulated and compared with the nominal performance, and the enhanced model could be used for sensitivity analysis. The method was applied to a highly complex helical surface that allowed for the adjustment of the optical power by rotation. We show that sensitivity analysis could be used to define acceptable tolerance budgets of the process.

## 1. Introduction

Additive manufacturing (AM) offers great freedom in design, a short lead-time, and the possibility of functional integration [1]. AM technologies can be divided into seven main categories [2,3], including 3D ink-jet printing. In 3D-ink-jet printing, ink droplets are deposited and cured, layer by layer, by UV illumination. The ink may be polymer or hybrid polymer (e.g., Ormocer (R)) based. Moreover, metal or ceramic nanoparticle-enhanced polymer inks continuously increase the application range of 3D-ink-jet printing. Ink rheological properties, piezo waveform, and nozzle shape are important parameters for droplet generation. Upon droplet impact on the substrate or on previously printed layers wetting behavior, droplet size, droplet overlap, droplet coalescence, and UV-curing strategy govern layer formation. A considerable amount of research has gone into developing an understanding of these complex interrelations between materials, equipment, and printed shapes. Though 3D printing was originally primarily used for prototyping, it has evolved to be used for the custom mass production of functional parts [4,5] and is used in several industrial fields, e.g., automotive, aerospace, and medical, as a well-established alternative to conventional manufacturing processes [6,7,8,9]. The potential of high-resolution 3D printing to produce parts with a high degree of design freedom makes this technology interesting for optical applications where an increasing use of optical freeform surfaces can be observed. Optical applications using freeform optical surfaces include automotive lighting [10,11], beam expanders [12], and ophthalmic implants [13,14,15]. The established processes to manufacture freeform optical components from optical polymers are the direct diamond turning of the optical component [16] and the diamond turning of a mold insert for precision injection molding [17]. High-resolution 3D printing, with its enormous flexibility in terms of shape variation, opens up great potential for creating new approaches and solutions for optical systems [18]. The manufacturing of optical components and, especially, freeform surfaces has to meet high requirements, e.g., the high fidelity of the optical surfaces. Macroscopic imaging freeform optics pose great challenges for high-resolution 3D inkjet printing with regard to shape fidelity [19]. There are only few companies mastering the above-mentioned complex set of 3D-inkjet printing to a degree that optical surface can be realized. This process know-how is their competitive advantage and thus, understandably, not disclosed to the public. For the performance prediction of the manufactured freeform optics in an optical system, the use of measurement data of the manufactured parts to enhance the optical model with respect to realistic representation of the optical surfaces as manufactured is a useful strategy [20]. Detailed comparisons of nominal freeform surfaces with tactile measurements of freeform surfaces produced by 3D inkjet printing allow for a better understanding of the 3D inkjet printing process and the derivation of process improvement strategies [21]. Though the term digital twin is versatilely used, there is a basic and common understanding in scientific and industrial publications that a digital twin is a virtual equivalent of a real system [22]. It maps physical objects and does not only describe physical objects but also optimizes physical objects based on models [23]. This article presents the development of a digital twin on the basis of the above-described approach to adapt the high-resolution inkjet printing process to a respective geometry with the objective of increasing the shape fidelity of optical surfaces. Our work was based on an enhancement of the simulation models with data from surface measurements. This allows for the detection of discrepancies in shape fidelity and for the direct determination of the influence of these discrepancies on the optical performance of a freeform component. Hence, the printing process can be adjusted in order to reduce or increase material accumulation or to adjust curing strategies, thus leading to higher-performance components. The organization of the paper is as follows: in Section 2, the digital twin and the process flow are developed. Section 3 introduces the optical system used as an example. The evaluation of the measurements leading to a height map of the difference surface between measured and nominal surface is presented in Section 4. Section 5 is about the model enhancement and the usage of the digital twin in sensitivity analysis. A discussion of the achievements closes this article.

## 2. Concept of Generating and Using the Digital Twin

**Figure 1:**
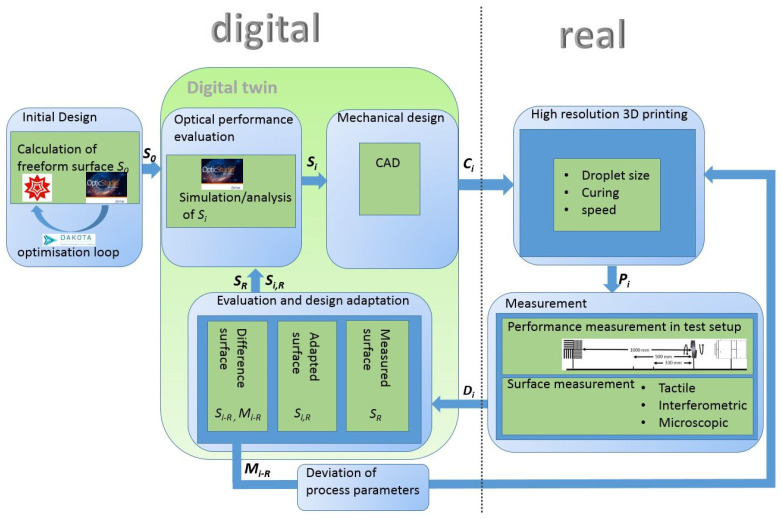
Digital twin and process flow.

The flow between the individual process steps of process parameter deviation and digital twin generation is shown in Figure 1. The first step was the creation of an initial optical design. The arbitrary freeform optical surface was calculated with the help of mathematical software. The mathematical development tool Mathematica [24] served as a tool in our example. To perform an optical analysis of the complete system model, the mathematical freeform surface was input to an optical simulation tool. In our example, OpticStudio [25] was used for this purpose. In order to perform an automatic analysis of the optical properties of different variants, a communication between the two tools was implemented based on the WSTP (Wolfram Symbolic Transfer Protocol) extension of OpticStudio. A design optimization of the initial design was realized by integration of the Single-Objective Genetic Algorithm (SOGA) of the Sandia Dakota (Design Analysis Kit for Optimization and Terascale Applications) Box [26]. The evolutionary algorithm controlled the optimization process of the optical freeform surface with respect to the specific system requirements. The optimized optical freeform surface *S*_0_ was the initial design for the process flow and was input in the optical performance evaluation where performance analysis was carried out on the basis of optical simulations. The surface *S_i_* (where the index *i* denotes the number of passes of this loop; the initial surface therefore has the index 0) was then transferred into mechanical design, where an optical component *C_i_* was generated by adding a thickness and support structures, e.g., substrate or mounting and alignment structures. Manufacturing complex structures requires an adaption of the design to the design rules of the involved processes, e.g., the manufacturing of the optics, or the assembly of the optical parts to an adjusted optical system.

A way to approach such adaptations and the final design for manufacture were described in [27]. The finalized CAD model contained both optical and mechanical structures in one comprehensive model and described the optical component *C_i_* that was digitally input to the printing process. Up to this transfer, the optical component was described entirely digitally. After the manufacturing process, a real part existed. The printing process took place on a high resolution 3D inkjet printer and was carried out by the Dutch company Luximprint [28]. The result of the printing process was a real optical part *P_i_* where the optical surface and the mechanical alignment structures were manufactured in a single process step [29]. In the next step of digital twin generation, measurements were carried out on the basis of the finished optical parts *P_i_*. The characterization of the optical properties of the printed optical component were conducted both in a laboratory setup to analyze the optical performance and by tactile surface measurement, using a Dektak V220 SI profilometer, to analyze the dimensional accuracy of the printing process. The resulting data *D_i_* of the measurement were again digital descriptions, now of the surface of the real manufactured part. On the basis of the measurement results, three different surfaces were generated: the surface *S_R_* of the real manufactured part, which was input into the optical performance evaluation to simulatively analyze the systems performance of the manufactured part; the surface *S_i,R_* that was an adaptation of the nominal design to different tolerance budgets of the manufacturing process and was also input to the optical performance evaluation for the sensitivity analysis of a manufacturing process with unknown tolerance distribution (see also Section 5); and the surface *S_i-R_* that was the difference between the nominal surface *S_i_* and the measured surface *S_R_*. On the basis of the difference surface *S_i-R_*, a spatially resolved height map *M_i-R_* of the differences between target and actual geometry was created by comparing the measured data with the nominal data of the freeform surfaces. This map was used to derive feedback to the printing process and to determine at which positions adjustments to material deposition were necessary. With this information, the printing process could be adjusted to the respective freeform surface by adapting printing parameters (printing speed, drop size, UV-illumination control for curing).

**Figure 2:**
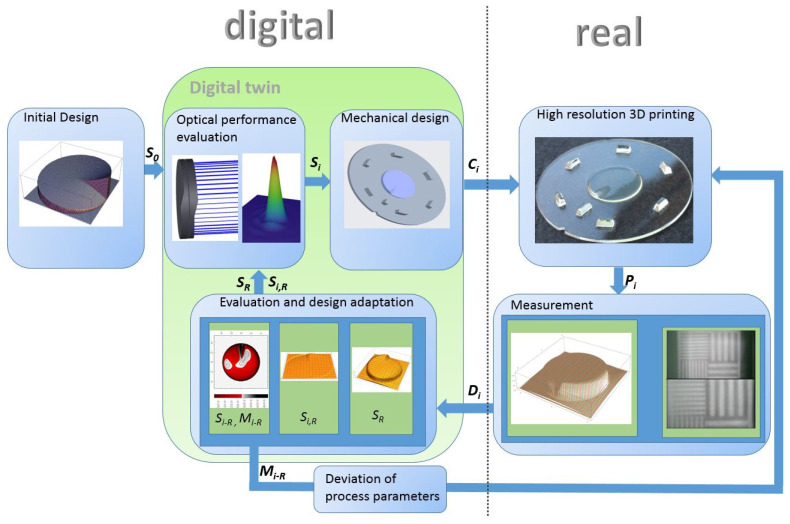
Simplified visual illustration of digital twin and process flow.

In Figure 2, exemplary results of the individual process steps are shown to illustrate the flow diagram in Figure 1. The initial optics design is depicted in superelevated representation, and the optical performance evaluation is presented by a raytracing section and a map of the point spread function. In the mechanical design, the underlying substrate is shown, as are the alignment and centering structures of the optical component. A detailed discussion of the individual alignment structures can be found in [29]. The high-resolution 3D ink-jet printed optics part acted as input to measurements. Imaging and surface measurements at the laboratory setup were conducted. Here, the measurement and imaging results of the tactile measurement are shown. The evaluation step shows the resulting measured surface, the adapted surface, and the height map. On the basis of these data, adjustments to the printing process, as well as input in the optics design process to enhance the systems model, were derived. Both ways led to creation of a digital twin that allowed us to simulatively evaluate the system performance of the manufactured parts and derive fabrication process improvements.

## 3. Optical System Used as Example

The example used to illustrate creation and application of the digital twin was a varifocal optics consisting of two optical freeform surfaces that were helical in shape with a radius of curvature changing azimuthally [30]. The freeform surfaces are described by Equation (1) [31].

(1) $$z\left(r,\alpha \right)=\frac{A\;}{2}r^{2}\alpha \;A:\;\mathrm{form}\;\mathrm{function};\;r=\;\sqrt{x^{2}+y^{2}\;};\;\alpha :\;\mathrm{azimuth}$$

**Figure 3:**
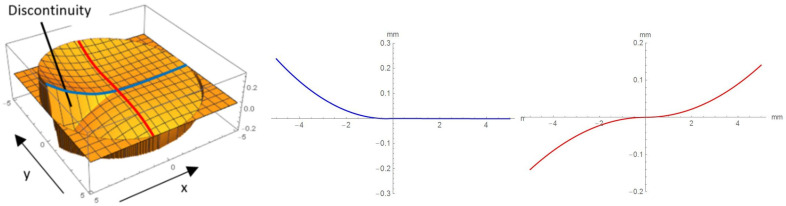
Left: Superelevated geometry of a lens surface with lines of intersection parallel to the x-axis (blue) and parallel to the y-axis (red). Middle: Profile parallel to x-axis along the blue intersection path. For positive abscissae, the ordinates are zero and the curve directly superimposes the x-axis. Right: Profile parallel to y-axis along the red intersection path [21].

**Figure 4:**
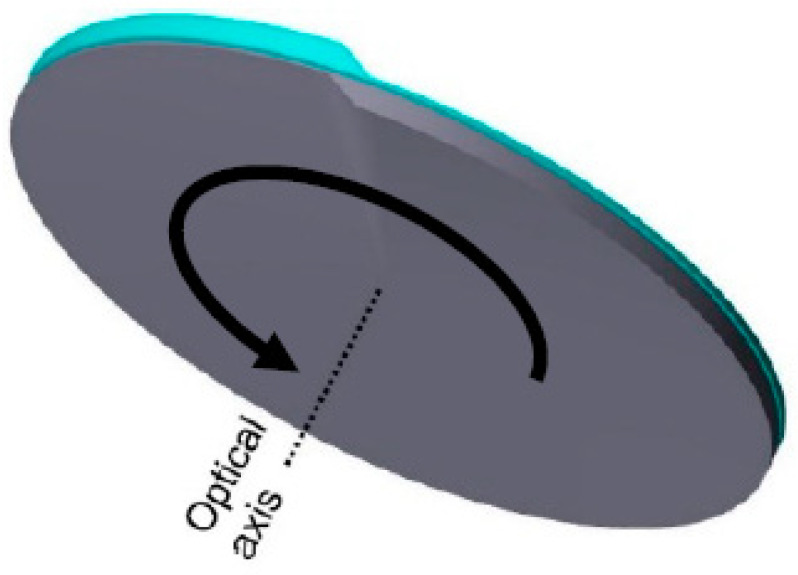
Rotation optics, consisting of two lens bodies with surfaces of azimuthal curvature dependence arranged coaxially to the optical axis and their plane surfaces facing each other [27].

The curvature of the lens bodies depends on the form factor *A* and the azimuth *α*, hence featuring a discontinuity at the transition from *α* = 2 π to *α* = 0. The left of Figure 3 shows a helical surface calculated with Equation (1): the surface curvature varied from convex to concave, starting with the discontinuity (transition between 2 π to 0) following the azimuth clockwise. The profiles of the surface parallel to the x-axis, along the blue intersection (Figure 3, mid column) and parallel to the y-axis along the red intersection path (Figure 3, right) illustrate the surface sag, which indicates the displacement of the surface along the optical axis (z-axis) of the surface at distance $r=\sqrt{x^{2}+y^{2}}$ from the axis. Since discontinuity could seriously affect imaging quality, it has to be obscured by a diaphragm to prevent any disturbing effects in imaging, e.g., scattering. A combination of two lens parts featuring such helical surfaces arranged in sequence perpendicular to the optical axis would possess a constant refractive power over the complete aperture. The refraction power could be tuned continuously by mutually rotating the lens bodies around the optical axis (see Figure 4). This optical system is multifocal, with two sectors with different individually tunable refraction powers [21,27].

Freeform surfaces like this with an azimuthal change of curvature and a discontinuity place very high demands on the manufacturing processes.

## 4. Evaluation of the Surface Measurements

The first four steps in the process flow (see Figure 1), namely initial design, optical performance evaluation [27], mechanical design [29], and printing [21], have been described elsewhere in detail. In this article, we focus on analyzing surface measurements of the freeform surface and on the derivation of the height difference map between nominal and printed geometry to determine at which positions adjustments of material deposition in the printing process were required. This information could be further used to derive process parameters to directly adjust the printing process to a respective freeform surface by adapting, e.g., printing speed, drop size, and UV-illumination control for curing.

**Figure 5:**
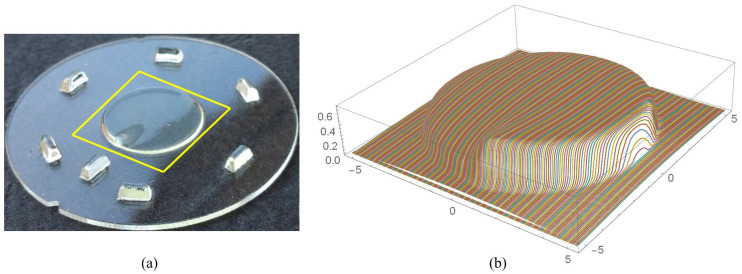
(**a**) Measuring window and (**b**) superelevated 3D-plot of the 210 measurement profiles.

Tactile surface measurements were conducted with a Dektak V220 SI profilometer. The measuring window had a dimension of 10.5 mm × 10.5 mm and encompassed the entire optical aperture (see Figure 5a). In the measurement window, 210 profile lines with 5250 measuring points each were recorded. The path of the measuring tip was chosen to be parallel to the surface discontinuity of the rotation optics. Figure 5b shows a superelevated 3D-plot of the 210 measurement profiles, each containing 5250 data points.

**Figure 6:**
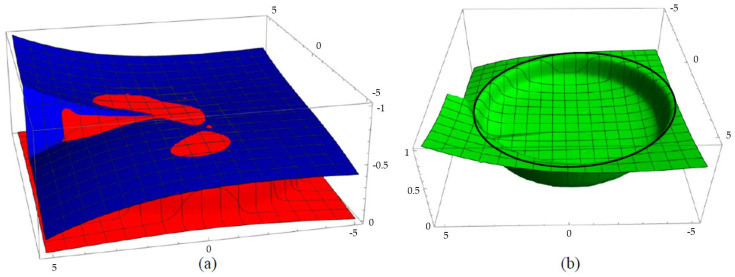
(**a**) Overlay of nominal surface (blue) and measured surface (red); (**b**) difference surface. The black circle indicates the nominal aperture.

**Figure 7:**
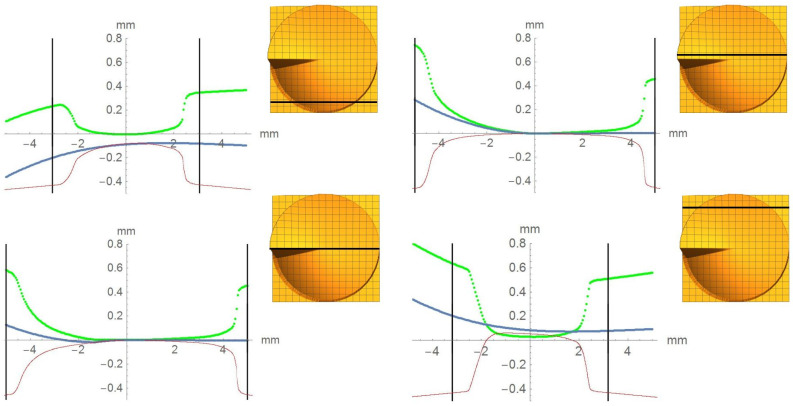
Measured profile (red), nominal profile (blue), and difference profile (green) inside the nominal aperture (solid black and perpendicular lines). Insets: position of the respective profile on the surface.

Application-specific manufacturing tolerances could be derived from the difference surface *S_i-R_* of the nominal freeform surface and the measurement data. Figure 6a shows the overlayed plots of the nominal freeform surface (blue) and the measurement data (red). It is obvious that too little material was printed in the edge areas of the aperture and too much material was printed in the central area and near the discontinuity. The penetration surfaces of the measurement data are clearly seen by the red islands in the blue nominal surface, indicating a surplus of material. The difference between measured and nominal surface is depicted in Figure 6b. Regarding the nominal aperture, indicated by the black ring, it is obvious that the printed optics did not entirely fill the aperture. The discrepancy between manufactured and nominal surface was evaluated by a comparison of the profiles. Figure 7 shows selected profiles spanning the entire measurement window.

**Figure 8:**
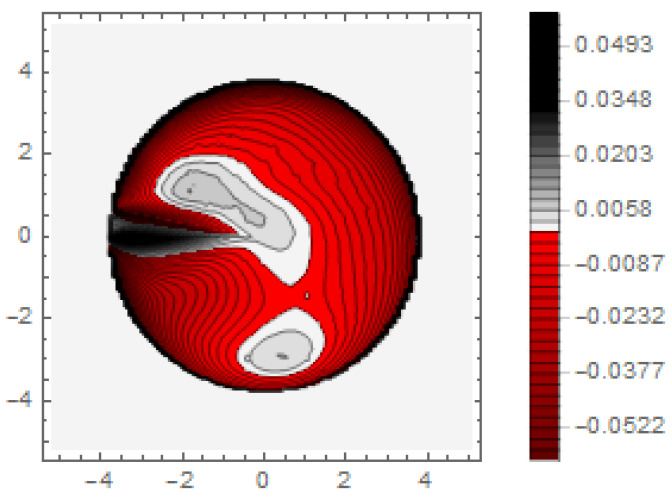
Height map representation of the difference surface.

The figure shows nominal profiles (blue), measured profiles (red), and difference profiles (green) inside the limits of the nominal aperture (perpendicular solid black lines). Considering Figure 7, it is apparent that the printed surface did not extend over the nominal lens aperture: the red curves depicted in Figure 7 dropped to substrate level far inside the nominal aperture. The evaluation showed a lens aperture reduced by about 20%. This shape deviation at the edge of the aperture could be corrected directly by adjusting the allowance in the process data. The compensation of the lateral shrinkage for such structures could lead to a design aperture of 125% of the nominal value [21]. Furthermore, strong form deviations could be observed, and these were most pronounced at the transition from convex to concave. These form deviations must be corrected by an adapted control of the high-resolution 3D printing process. To get information of shape deviation over the entire optical aperture, the difference surface is represented as a spatially resolved height map *M_i-R_* (Figure 8). The scale of the height map directly shows the tolerance range with which the respective design could be manufactured by the specific process. Regarding the given case, the tolerance of the surface accuracy was in the range of ±50 µm. However, the height map also quantitatively shows the difference between nominal and measured surface at every surface position. This directly gives information regarding in which areas more and in which areas less material must be deposited; hence, this was a quantitative measure of the surface accuracy of the printing process. The quantitative information of the height map could be used to adjust the parameters of the high-resolution printing by adapting printing speed, drop size, and UV-illumination control for curing and to define an allowance for compensation.

## 5. Model Enhancement and Digital Twin

To be able to analyze the printed parts by means of optical simulation, an enhancement of the optical model was implemented. The model enhancement was conducted by integrating the measuring surface data *S_R_* into the simulation model [20]. Hence, a performance evaluation of the printed part was enabled in the digital space. This approach also enabled a comparison of the printed parts with the nominal surface description. Table 1 shows a comparison of imaging analysis between the nominal surface *S*_0_ and the printed optics (represented by the measuring surface data *S_R_*). The MTF (modulation transfer function), a well-established measure of optical image quality, and the geometrical imaging of a bar pattern are presented, each for three adjustments of the varifocal optics: 1, 2, and 3 dpt (where dpt is the unit symbol for diopter which is the unit of optical power: 1 diopter = 1 m^−1^). The first row shows the simulation results of the nominal surface, and the second row shows the simulation results for the optical surface reconstructed from measurement data. As shown above, the achieved dimensional accuracy corresponded to a tolerance of ±50 µm. The first row of Table 1 shows the performance of the nominal rotation optics. The MTF, depicted up to a spatial frequency of 100 lp/mm, is smooth and resulted in a value of around 0.4 at 100 lp/mm for each of the three adjustments. This value could be seen as the contrast with which details with a spatial frequency of 100 lp/mm could be resolved by the optical system. Results are presented underneath the MTF of the simulated imaging. The clear and sharp imaging of the bar pattern confirms the good imaging behavior of the nominal system. The second row shows the same analysis for the measured data representing the manufactured part. The MTF collapsed at around 11 lp/mm (please take notice of the different scale of the spatial frequency axis-in case of the measured data; the MTF is depicted only up to 50 lp/mm). The geometric image analysis also reflects the MTF: the harsh collapse of MTF led to a blurred image of the bar pattern. Ghost reflections, resulting from non-matching surfaces, could also be observed.

The enhanced model on the basis of surface *S_i,R_* could be further used as a digital twin to predict system performance due to realistic and application-specific manufacturing properties. As defined in Section 2, the surface *S_i,R_* is an adaptation of the nominal surface to different tolerance budgets of the manufacturing process. The approach to this is to confine the difference between nominal and measured surface to a specific tolerance range. By adding this confined deviation spatially resolved to the nominal surface, a model of a manufactured optics was generated for these specific manufacturing tolerances. Hence, based on measurement data, a new optical surface was synthetically created. In this way, the effect of different process parameters and processing strategies on component performance could be studied in digital space without physically manufacturing a large number of parts.

Using the digital twin to predict optical performance for different tolerance regimes allows for a sensitivity analysis to determine acceptable manufacturing tolerances in order to achieve specific performance targets. Table 2 shows the simulation-based optical analysis of a rotation optics constrained with a manufacturing tolerance of ±10 µm. A comparison of the simulation results of the imaging quality of the adapted model (Table 2) with that of the measured data (row 2 in Table 1) allows for the performance evaluation of the two tolerance regimes: ±50 µm as manufactured and ±10 µm as adapted. Regarding the MTF, an improvement was mainly seen in the 1 dpt adjustment. The improvement of the MTF for the 2 and 3 dpt adjustments are not as eye-catching. Regarding the image analysis underneath the MTF, the improvement in image quality is more obvious. The details in the bar pattern are resolved, the contrast is higher, and the ghost images are reduced.

The sensitivity analysis of anticipated manufacturing tolerances of ±10 µm resulted in the expected system performance and thus allowed for the definition of an acceptable range of manufacturing tolerances depending on system performance.

## 6. Discussion

The article demonstrates a process flow with the aim of enhancing high resolution 3D inkjet printing to manufacture optical freeform surfaces by means of a digital twin. Besides its obvious advantages like fast and cost-efficient fabrication, 3D-inkjet printing offers the possibility to manufacture almost arbitrary shapes. To enable the inkjet printing process to manufacture freeform surfaces in optical quality, we proposed a process flow between optical performance evaluation, mechanics design, high resolution inkjet-printing, measurement, and an evaluation and design adaptation with a feedback to be suited to control the printing process. Regarding the example of rotation optics in this article, we focused on the evaluation of the measurement data and the creation of a digital twin. First, the evaluation of the measured data resulted in a height map that quantitatively indicated the difference between the printed surface shape and the nominal shape. This information was available in spatial resolution and, hence, could be used to derive process parameters, e.g., printing speed, drop size, and UV-illumination control for curing for the specific freeform surface, to control the printing process so that material deposition and printing parameters could be adjusted. Second, the enhanced model was used to simulate the performance of the manufactured part and compare it to the performance of the nominal shape. The enhanced model was further used as a digital twin for sensitivity analysis regarding the interaction between process and part when manufacturing tolerances are not known. Additionally, this approach allowed for the definition of the minimum dimensional accuracy for a given surface shape to maintain the required system performance.

The surface roughness of printed components could also have an impact on optical performance. A measurement of surface roughness could be integrated into the process flow, as shown in Figure 1, in the measurement box and evaluated by means of a digital twin. The consideration of the surface roughness of the printing process with the aim of adjusting the process parameters to achieve a higher surface quality is a concern of future work.

## Figures and Tables

**Table 1:** The modulation transfer function (MTF) and geometric image analysis of three different adjustments of the rotation optics (1, 2, and 3 dpt) for the nominal surface (first row) and the measured data (second row).

1 dpt	2 dpt	3 dpt
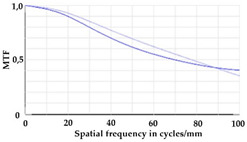	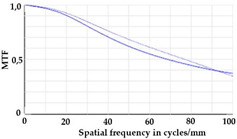	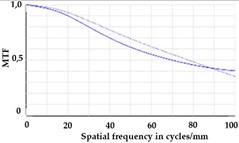
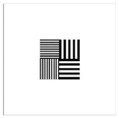	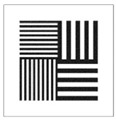	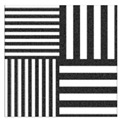
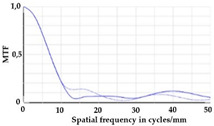	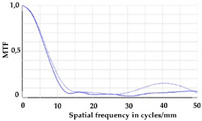	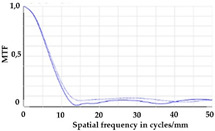
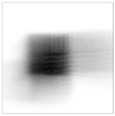	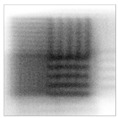	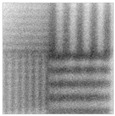

**Table 2:** Modulation transfer function (MTF) and geometric image analysis of three different adjustments of the rotation optics (1, 2, and 3 dpt) for the nominal surface constraint with a manufacturing tolerance of ±10 µm.

1 dpt	2 dpt	3 dpt